# The Journal of Comparative Medicine

**Published:** 1881-01

**Authors:** 


THE JOURNAL OF COMPARATIVE MEDICINE enters its second volume under the best auspices. Help has been promised it, in the form of contributions and subscriptions, from various quarters, and there is no reason to doubt its success in the new field that has been chosen.

It will be the central object of the JOURNAL to discuss and contribute to the knowledge of the diseases of animals, especially of the horse.

It has been thought best to substitute the name JOURNAL for "ARCHIVES," since the former title better expresses the character and scope of the periodical. It will contain news and discussions, and will be something more than a mere repository. The necessity for such a change would be absolute, in case the JOURNAL should be more frequently issued.

